# An Unusual Case of Ileitis and Partial Small Bowel Obstruction Secondary to Mesh Erosion After Totally Extraperitoneal Inguinal Hernia Repair

**DOI:** 10.1155/crgm/3047912

**Published:** 2025-05-05

**Authors:** Pranesh de Silva, Joseph Do Woong Choi, Craig Lynch, Stephen Pillinger, Saurabh Gupta, Praveen Ravindran

**Affiliations:** ^1^Department of Colorectal Surgery, Sydney Adventist Hospital, Wahroonga, New South Wales, Australia; ^2^College of Health and Medicine, Australian National University, Australian Capital Territory, Canberra, Australia; ^3^Faculty of Medicine and Health, The University of Sydney, Sydney, New South Wales, Australia

**Keywords:** ileitis, laparoscopic extraperitoneal inguinal hernia repair, mesh erosion, small bowel obstruction

## Abstract

The objectives were to highlight that: (1) mesh erosion related partial small bowel obstruction after laparoscopic totally extraperitoneal (TEP) inguinal hernia repair (IHR) as an uncommon complication can clinically and radiologically mimic ileocolic Crohn's disease in young adults; and (2) implore clinicians to consider a broad set of differential diagnosis and prompt involvement of other subspecialties, especially if preliminary investigations and treatment yield minimal results. The authors report a 34-year-old male who presented with computed tomography (CT) findings of ileitis, which was initially investigated for Crohns' disease. Due to persisting abdominal pain and negative initial investigations, he underwent a laparotomy demonstrating secondary mesh migration with erosion into distal ileum requiring bowel resection, 2.5 years after an uneventful laparoscopic right TEP IHR. The patient made an uneventful postoperative recovery and at 6 weeks follow-up, he had resolution of abdominal pains, and normal bowel function.

## 1. Introduction

Inguinal hernia repair (IHR) is one of the most common general surgical procedures with approximately 20 million surgeries performed annually worldwide. The incidence of inguinal hernia in Australia is approximately 194 per 100,000 of population, mainly affecting males [1]. There has been an increased uptake of minimally invasive surgery away from open IHR over the last decade. The two laparoscopic approaches for IHR are transabdominal preperitoneal procedure (TAPP) and a totally extraperitoneal (TEP) approach [2]. A rare complication following laparoscopic TEP IHR includes bowel obstruction with an overall incidence rate of 0.07% [3].

The authors present a unique case of a patient who was being worked up for Crohn's related ileitis, however a partial small bowel obstruction secondary to mesh erosion from a recent laparoscopic TEP repair was unexpectedly found.

## 2. Case Presentation

**Figure 1:**
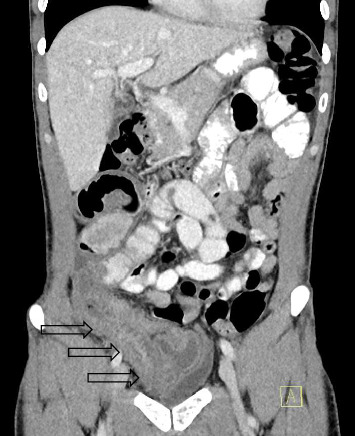
Computed tomography (CT) scan demonstrating a long segment of distal small bowel wall thickening with mucosal hyperenhancement consistent with ileitis (arrows).

**Figure 2:**
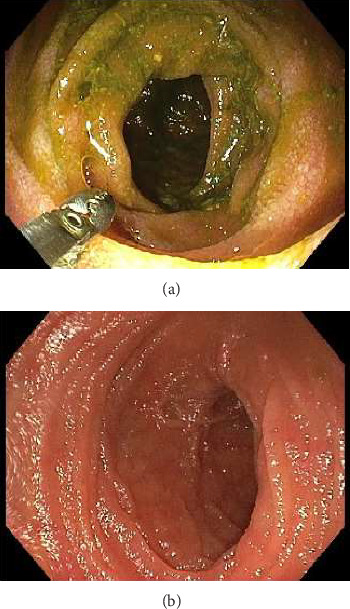
Colonoscopic pictures of normal ileal mucosa at the ileocaecal valve (a) and 60 cm proximal to the ileocaecal valve (b).

A 34-year-old Caucasian male presented to the emergency department with a 4 week history of right lower quadrant pain. This was associated with intermittent per rectal bleeding. He denied nausea, vomiting, diarrhoea, change in bowel habits or significant weight loss. He was treated at another hospital 3 weeks prior with intravenous antibiotics for presumed infective enteritis. The background history was significant for an uncomplicated laparoscopic right TEP IHR with lightweight polypropylene mesh 2.5 years ago, and depression on Sertraline. On examination, the observations were normal, afebrile. Abdomen was soft, with tenderness in the right iliac fossa without peritonism. Biochemistry demonstrated a white cell count (WCC) 10.9 (range: 4–11 × 10^9^/L), haemoglobin (Hb) 147 (range: 130–180 g/L), c reactive protein (CRP) 4.3 (range: 0–5 mg/L) and erythrocyte sedimentation rate (ESR) 2 (range 1–20 mm/h). Faecal calprotectin, faecal viral polymerase chain reaction (PCR) for norovirus, rotavirus, adenovirus, sapovirus, astrovirus, stool microscopy/culture/sensitivities (MCS), *Clostridium difficile* toxin PCR, Yersinina 3 and pseudotuberculosis antibodies and QuantiFERON gold tests were negative. A CT scan demonstrated a long segment of distal small bowel wall thickening with perienteric fat stranding (Figure 1, arrows) and mildly prominent ileocolic lymph nodes consistent with ileitis. There was no small bowel dilatation proximally, or other complicating features. He was initially admitted under gastroenterology with a high index of suspicion for Crohn's disease, given his age. Intravenous hydrocortisone 100 mg four times a day was initiated, as was a clear fluid diet. A colonoscopy with intubation of 60 cm into ileum performed a few days later demonstrated normal ileal (Figure 2) and colonic mucosa, and internal haemorrhoids. Biopsies of the ileum returned normal.

**Figure 3:**
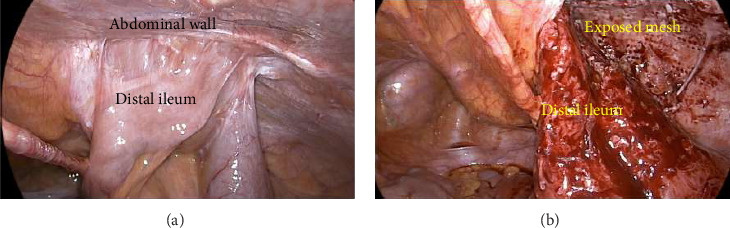
Intraoperative views of dense distal small bowel adhesion onto the mesh (a) and after adhesiolysis demonstrating erosion of mesh onto this small bowel segment that was subsequently resected (b).

As he remained tender with unexplained ileitis for 4 weeks, and after 5 days of unsuccessful medical management, he underwent a diagnostic laparoscopy. This demonstrated extensive small bowel adhesions distally secondary to mesh contact on bowel from previous right TEP IHR leading to partial small bowel obstruction (Figure 3(a)). Conversion to lower midline laparotomy was performed with extensive adhesiolysis. The small bowel was gently peeled off from the mesh, however due to the mesh erosion there was extensive small bowel injury (Figure 3(b)). There was no evidence of hernia recurrence. As such, 40 cm of the involved small bowel was resected with a side-to-side anastomosis using a Covidien GIA80 stapler (Medtronic, Minneapolis, USA). The mesh was explanted from the preperitoneal plane as much as possible medially, however the mid to lateral portions were unable to be safely removed due to the proximity of the common iliac vessels. The latter component was covered with a peritoneal flap and bladder to exclude the remaining mesh from the intra-abdominal viscera. The histopathology of the small bowel demonstrated tissue reaction to foreign body material without malignancy, consistent with mesh erosion. Postoperatively, the patient made an uneventful recovery with normal bowel function and enteric diet. He was discharged 8 days after surgery, and at 6 weeks follow-up, had resolution of abdominal pains.

## 3. Discussion

The case highlights a partial small bowel obstruction secondary to mesh erosion as a rare complication after a recent laparoscopic TEP IHR 2.5 years ago [3, 4]. In a study involving 17,587 patients who underwent laparoscopic IHR, intestinal obstruction occurred 0.06% postoperatively, highlighting our patients' unusual presentation [5]. It is suggested that a peritoneal defect can lead to direct contact between the mesh and intra-abdominal organs resulting in adhesions, mechanical obstruction, mesh erosion and fistula formation [6] Al-Zubaidi et al. reported case in which the bowel became entrapped in a peritoneal defect after laparoscopic TEP IHR [7]. It was suggested that the deflation of the preperitoneal space intraoperatively may have caused bowel to enter the defect [8]. Additionally, Aggarwal et al. highlighted a case of mesh erosion into small bowel and urinary bladder, requiring a small bowel resection 21 months after laparoscopic TEP IHR [6]. They suggested that primary mesh migration may have resulted in this presentation, due to inadequate fixation or external displacing forces [6, 9]. Strenuous activity including bending and hip flexion in the immediate postoperative period during which the mesh has the greatest likelihood of dislodging may be a causative factor. In addition, secondary migration may have also occurred as a consequence of a foreign body reaction leading to erosion through anatomic planes. Weakening of the small bowel wall and subsequent erosion of viscus may also occur due to sharp cut edges of mesh leading to an inflammatory reaction [9, 10]. In our case, no defect in the peritoneum was reported from the original repair. However, the authors suspect that the direct contact of mesh onto small bowel found on diagnostic laparoscopy may have been related to either missed peritoneal defect, or folds in the mesh due to insufficient flat placement against the abdominal wall due to inadequate dissection of the landing zone may have precipitated secondary mesh migration leading to erosion through the peritoneum onto adjacent ileum leading to an unusual CT findings of ileitis.

Other cases of small bowel obstruction secondary to mesh contact highlighted a variety of CT findings. Ali-Zubaidi et al. demonstrated CT findings of an extraperitoneal incarcerated small bowel obstruction with proximal dilated jejunal loops and a transition point at the peritoneal breach [7]. Similarly, Berney performed a diagnostic laparoscopy and reduced a preperitoneal hernia 5 days after a laparoscopic TEP IHR as CT findings highlighted several small bowel loops down to a transition point in the right iliac fossa [11]. This complication occurred even after the divided sac from the index operation was closed with a pre-tied PDS endoloop (Endoloop Ligature PDS II, Ethicon Endo-Surgery, Somerville, NJ).

There are no studies to date that have investigated rates of mesh erosion with the use of different type of mesh material after laparoscopic TEP repair. This is likely due to the rarity of the complication. Mesh erosion rate was found to be higher with the use of synthetic (0.5%) or with the use of synthetic and biological mesh when used together (1.4%) versus biological mesh (0%) after laparoscopic rectopexy [12].

Diagnosis of mesh erosion can be challenging due to the scarcity of occurrence as well as the nonspecific symptoms of presentation [13]. Clinical signs of mesh erosion range from abdominal pain to sepsis, and often vary due to the organ involved [14]. It is suggested that the combined use of imaging studies such as with CT and a high index of clinical suspicion will diagnose mesh erosion regardless of the time elapsed from the primary repair [13]. In our case, the CT demonstrated distal small bowel wall thickening with perienteric fat stranding and prominent ileocolic lymph nodes suggestive of ileitis. An initial suspicion for Crohn's disease or infective gastroenteritis was ruled out after biochemistry and colonoscopy were normal, thus prompting operative management. Upon identification of mesh related small bowel obstruction, surgical management is the cornerstone of management [13]. Minimally invasive surgery with laparoscopy could be attempted in entirety experienced hands, however in the presence of dense adhesions from mesh erosion and the need for small bowel resection as was in our case, an open approach or conversion may be more appropriate.

## 4. Conclusion

This case illustrated an unusual presentation of secondary mesh migration with distal small bowel partial obstruction and erosion in light of the radiological findings of a long segment of distal ileitis (Figure 1). Clinicians should maintain a high index of suspicion after laparoscopic TEP IHR if any unusual abdominal symptoms persist, despite medical management. If radiological findings are nonspecific along with negative biochemical and/or endoscopic investigations, diagnostic laparoscopy should be offered promptly.

## Data Availability

The data that support the findings of this study are available on request from the corresponding author. The data are not publicly available due to privacy or ethical restrictions.
